# Altered contractile responses to angiotensin II and cholinergic stimulation in colonic circular smooth muscle from patients with diverticular disease

**DOI:** 10.3389/fphys.2026.1991333

**Published:** 2026-10-14

**Authors:** Mantas Malinauskas, Justas Žilinskas, Darius Stukas, Kristina Rysevaite-Kyguoliene, Deividas Nekrosius, Henrikas Paužas, Andrius Macas, Anna Casselbrant

**Affiliations:** 1 Institute of Physiology and Pharmacology, Lithuanian University of Health Sciences, Kaunas, Lithuania; 2 Department of Surgery, Lithuanian University of Health Sciences, Kaunas, Lithuania; 3 Institute for Digestive Research, Lithuanian University of Health Sciences, Kaunas, Lithuania; 4 Institute of Anatomy, Lithuanian University of Health Sciences, Kaunas, Lithuania; 5 Department of Anesthesiology, Lithuanian University of Health Sciences, Kaunas, Lithuania; 6 Department of Surgery, Institute of Clinical Sciences, Sahlgrenska Academy, University of Gothenburg, Gothenburg, Sweden

**Keywords:** angiotensin II, colon, diverticular disease, renin–angiotensin system, smooth muscle contractility

## Abstract

**Background:**

Abnormal circular smooth muscle contractility is considered a key factor in the pathogenesis of diverticular disease (DD), contributing to increased intraluminal pressure and diverticulum formation. Altered cholinergic responsiveness of colonic smooth muscle has previously been reported in DD. Although angiotensin II (Ang II) regulates gastrointestinal smooth muscle contraction through angiotensin type 1 (AT1R) and type 2 (AT2R) receptors, its role in DD remains poorly understood. The aim of the present study was to investigate alterations in contractile responses to Ang II and cholinergic stimulation in the circular muscle of the colon in DD.

**Methods:**

Colonic tissue was obtained from 16 patients with DD and 16 control patients undergoing colorectal surgery. Immunofluorescence was performed to determine the localization of AT1R and AT2R. Ang II levels in colonic muscular tissue were quantified by enzyme-linked immunosorbent assay (ELISA), AT1R and AT2R protein expression were evaluated by Western blotting, and their mRNA expression was assessed by RT-qPCR. Circular smooth muscle strips were subsequently studied using *in vitro* organ bath recordings to assess Ang II-induced contractile responses under basal conditions and following pharmacological blockade with losartan, tetrodotoxin (TTX), or the AT2R antagonist PD123319.

**Results:**

AT1R was predominantly localized in colonic smooth muscle cells, whereas AT2R was mainly detected in the myenteric plexus and nerve fibers. Compared with controls, DD tissues exhibited increased local Ang II levels and higher AT2R protein expression, accompanied by increased AT2R mRNA expression. Bethanechol-induced contractility was reduced in DD, whereas both peak and mean 5-min Ang II-induced contractile responses were enhanced. Losartan significantly reduced the mean 5-min Ang II-induced contractile response in both groups. However, both peak and mean responses remained significantly greater in DD than in controls following AT1R blockade. Neither TTX nor PD123319 significantly affected Ang II-induced contractions.

**Conclusions:**

The present study demonstrates altered colonic circular smooth muscle contractility in diverticular disease, characterized by enhanced responses to Ang II and reduced responses to cholinergic stimulation. The distinct muscular and neural distribution of AT1R and AT2R, respectively, suggests that altered local Ang II signaling, together with altered cholinergic regulation, may contribute to the pathogenesis of DD.

## Introduction

Diverticular disease (DD) includes diverticulosis, characterized by the presence of colonic diverticula, and diverticulitis, characterized by inflammation and/or infection of diverticula (Von Rahden and Germer, 2012). Diverticulitis may present with abdominal pain, fever, and altered bowel habits and can lead to complications such as perforation, abscess formation, fistula, or bowel obstruction (Bhatia and Mattoo, 2023). Although only approximately 4–15% of individuals with diverticulosis develop acute diverticulitis (Hawkins et al., 2020; Bhatia and Mattoo, 2023), the burden of symptomatic diverticular disease may be underestimated because its clinical manifestations, including abdominal pain and altered bowel habits, frequently overlap with those of irritable bowel syndrome (IBS) (Hawkins et al., 2020). Therefore, a better understanding of the mechanisms underlying the development and progression of diverticular disease, particularly the transition from uncomplicated diverticulosis to inflammatory diverticulitis, is essential for improving diagnosis, prevention, and treatment. Pathogenesis of colonic diverticula is regarded as a multifactorial process, involving dietary factors (low-fiber diet) (Burkitt et al., 1972; Aldoori and Ryan-Harshman, 2002; Aune et al., 2020), structural changes of the colonic musculature (Hellwig et al., 2014), and functional changes (motility disorder) (Bassotti et al., 2001; Hellwig et al., 2014; Pauza et al., 2019), which result in increased intraluminal pressure (Painter and Truelove, 1964; Painter et al., 1965). Genetic changes and aging are also a key factor (Spiller, 2015; Nehring and Przybyłkowski, 2025). Abnormal colonic motility is thought to be an early pathogenic event in diverticular disease that precedes structural changes, including thickening of the circular muscle layer and contracture of the taeniae coli (Hughes, 1969; Whiteway and Morson, 1985). Notably, functional abnormalities associated with diverticular disease predominantly involve the circular muscle layer, whereas the longitudinal muscle largely retains normal electrophysiological activity (Huizinga et al., 1999). The circular smooth muscle is primarily responsible for colonic segmentation, which has been proposed to generate localized intraluminal pressure and the pulsion force leading to mucosal herniation through weak points of the colonic wall during diverticulum formation (Painter et al., 1965). Acetylcholine, a key excitatory neurotransmitter in the gastrointestinal tract, primarily mediates smooth muscle contraction via M3 muscarinic receptors (Bharucha et al., 2010). Both the circular and longitudinal muscle layers exhibit reduced cholinergic innervation and upregulation of M3 muscarinic receptors in diverticular disease (Golder et al., 2003). Therefore, increased sensitivity to acetylcholine has been demonstrated predominantly in the circular muscle layer, indicating enhanced contractile responsiveness (Huizinga et al., 1999). Angiotensin II (Ang II) is another potent regulator of gastrointestinal smooth muscle contraction (Ewert et al., 2006; Patten and Abeywardena, 2017; Ferreira-Duarte et al., 2021; Esteves-Monteiro et al., 2022; Ha et al., 2022). Ang II acts via cell surface receptors subdivided into AT1 or AT2 type, characterized by using selective ligands (De Gasparo et al., 2000; Fändriks, 2011). However, its potential contribution to the altered contractile responses observed in diverticular disease remains unclear.

Considering the above, the aim of the present study was to investigate alterations in contractile responses to Ang II and cholinergic stimulation in the circular muscle of the colon in DD.

## Materials and methods

### Study participants

Control (Ctrl) tissue samples were obtained from patients undergoing colorectal resection for colorectal cancer who had no evidence of diverticulosis or previous episodes of symptomatic diverticular disease. Tissue specimens were obtained from patients who underwent surgery for symptomatic diverticular disease or in whom colonic diverticulosis was identified incidentally during colorectal surgery performed for another indication. In addition, the severity of diverticulosis was assessed based on the approximate number of colonic diverticula identified during preoperative colonoscopy and classified into three categories: 0–10, 10–100, and >100 diverticula.

Patients with irritable bowel syndrome, food intolerance, coeliac disease, Crohn’s disease, or ulcerative colitis were excluded from the study. Patients with arterial hypertension or receiving medications known to affect gastrointestinal motility or the renin–angiotensin system (RAS) were also excluded. These medications included β-blockers, diuretics, calcium channel blockers, angiotensin II receptor blockers (ARBs), angiotensin-converting enzyme (ACE) inhibitors, and α-blockers. Patients who had received neoadjuvant chemotherapy and/or radiotherapy before surgery were also excluded to avoid potential treatment-related effects on colonic tissue and contractile function.

The study was approved by the Kaunas Regional Biomedical Research Ethics Committee, Kaunas, Lithuania (Permission number: BE-2-37).

### Preparation of surgically resected colonic tissue

Full-thickness colonic specimens were collected in the operating room from macroscopically normal regions of the colon in patients with colorectal cancer and from peridiverticular tissue in patients with diverticular disease. Diverticula exhibiting altered colonic wall architecture due to transmural mucosal and submucosal outpouching, inflammation, or fibrotic scarring were excluded from tissue sampling. Immediately after excision, specimens were placed in pre-aerated (95% O_2_, 5% CO_2_) Krebs–Henseleit solution at 4 °C (118 mM NaCl, 4.7 mM KCl, 1.2 mM MgSO_4_, 1.2 mM NaH_2_PO_4_, 25 mM NaHCO_3_, 2.5 mM CaCl_2_, 11 mM glucose). Following resection, the mucosal and submucosal layers were carefully separated from the underlying muscularis propria by sharp dissection. The isolated muscularis propria was divided into three portions: one was snap-frozen in liquid nitrogen and stored at −80 °C for protein analysis, one was fixed for subsequent immunofluorescence analysis, and the remaining portion was used immediately for *in vitro* contractility experiments.

### Immunofluorescence

Tisue samples obtained from patients were washed in 0.1 M phosphate-buffered saline (PBS) and then fixed with 4% paraformaldehyde solution (PFA) in 0.1 M PBS (pH=7.4) for 2–3 hours, depending on the sample size. After, again washed in PBS, then immersed in 30% sucrose in 0.1M PBS containing 0.1% sodium azide in 4 °C for 24 hours. Following cryoprotection, intestinal tissue was frozen at -30 °C using a tissue-freezing medium (Triangle Biomedical Sciences, USA) for sectioning. Next, tissues were sectioned into 18 µm slices using a cryomicrotome HM 560 (Microm, Germany) at -22 °C, mounted onto Superfrost Plus microscope slides (Menzel Glaser, Germany), and dried at room temperature for 30 min. For immunohistochemical permeabilization, sections were incubated for 40 min. in a solution containing 9% dimethyl sulfoxide (DMSO) and 1% Triton X-100 in 0.1M PBS. After 3x10 min washing in PBS, sections were applied with 5% normal donkey serum (NDS) (Jackson ImmunoResearch Laboratories, West Grove, PA, USA), immunohistochemical procedures were continuing applying primary antibodies: rabbit polyclonal anti-AT2R (Thermo Fisher Scientific, Rockford, IL, PA3-210; 1:500), goat polyclonal anti-AT1R (Novus Biologicals USA, NB100-57073; 1:500), mouse monoclonal anti-HuC/HuD (Thermo Fisher Scientific, Rockford, IL, A21271; 1:500), mouse monoclonal anti-PGP 9.5 (Thermo Fisher Scientific, Rockford, IL, 480012; 1:500), mouse monoclonal anti-alpha smooth muscle actin (Abcam, UK, ab7817; 1:500), overnight at 4 °C. After washing in PBS, secondary antisera: donkey anti-rabbit (Millipore, Burlington, MA, AP182C, conjugated with Cy3 fluorochrome), donkey anti-mouse (Millipore, Burlington, MA, AP192F, conjugated with FITC fluorochrome) and donkey anti-goat (Millipore, Burlington, MA, AP180C, conjugated with Cy fluorochrome) were applied on the sections. Finally, specimens were washed three times for 5–8 min in 0.1 M PBS mounted with a Vectashield Mounting Medium (Vector Laboratories, Inc., Burlingame, CA, USA), cover slipped and sealed with clear nail polish. Primary antibody (negative) controls were processed in parallel to inspect signal specificity. Preparations were imaged using a confocal laser scanning microscope LSM 700, with the software package ZEN 2010B SP1 (Carl Zeiss, Jena, Germany).

### Gene expression analysis

#### RNA extraction from muscular tissues

Frozen tissue samples (approximately 25–30 mg) were homogenized using MagNA Lyser (Roche, Germany) prior to RNA extraction. RNA extraction was performed using RNeasy Mini Kit (Qiagen, Germany, Cat. No. 74104) according to the manufacturer’s instructions. RNA quality and concentration were evaluated using a NanoDrop 2000 spectrophotometer (Thermo Fisher Scientific, USA). The A260/A280 absorbance ratio of total RNA ranged from 1.9 to 2.1, indicating high purity.

#### Complementary DNA synthesis

Reverse transcription (RT) was performed using the High-Capacity cDNA Reverse Transcription Kit (Thermo Fisher Scientific Baltics, Lithuania, cat. no. 4368814) in a 20 μL reaction volume with 0.5 μg total RNA as input, according to the manufacturer’s protocol. The amplification reaction was performed in a thermal cycler (Applied Biosystems, USA) using the following cycling profile: 25 °C for 10 minutes, 37 °C for 120 minutes, and 85 °C for 5 minutes. After the reaction, cDNA samples were stored at -80 °C.

#### Quantitative real-time polymerase chain reaction

Quantitative RT-PCR (qRT-PCR) analysis was performed using an ABI 7500 fast real-time PCR system (Applied Biosystems, USA) to evaluate expression changes of AT1R and AT2R in colonic muscular tissue. The housekeeping gene GAPDH was used as an endogenous control, and a no-template sample was included as a negative control. All assays were performed in triplicate. The total volume of the qRT-PCR reaction was 10 μl: 5 μl TaqMan Fast Advanced Master Mix 2X (Applied Biosystems, USA, Cat. No. 4444557), 0.5 μl TaqMan™ Gene Expression Assay 20X (Applied Biosystems, USA, Cat. No. 4331182), 1 μl cDNA template and 3.5 μl nuclease-free water. TaqMan PCR assays were used for the detection of GAPDH, AT1R, AT2R: Hs99999905_m1, Hs00241341_m1, Hs00169126_m1 respectively. PCR parameters were used as recommended for the rapid cycling mode: enzyme activation at 95 °C for 20 s, followed by 40 cycles of 3 s at 95 °C for denaturation and 30 s at 60 °C for annealing. Fluorescence data were converted to threshold cycle (Ct) values and analyzed using the comparative 2^-^ΔΔCt method, with relative mRNA expression presented as fold change (2^-^ΔΔCt) to clearly depict relative changes in AT1R and AT2R mRNA expression.

### Protein analysis

#### Lysate preparation

Colonic muscle samples stored at −80 °C were thawed on ice and weighed. Tissues were rinsed with ice-cold phosphate-buffered saline (PBS), finely minced, and mechanically homogenized in Lysing Matrix D tubes (MP Biomedicals, Santa Ana, CA, USA) containing ice-cold RIPA lysis buffer supplemented with protease inhibitors (Thermo Fisher Scientific, Waltham, MA, USA). Homogenization was performed using a Bead Mill MagNA Lyser (Roche, Basel, Switzerland) with three cycles at 6000 rpm, each separated by a 15-s cooling interval. The lysates were incubated on ice for 30 min with intermittent vortexing and centrifuged at 10,000 × g for 10 min at 4 °C. The resulting clarified supernatants were aliquoted and stored at −80 °C until further analysis.

#### Quantification of protein levels

The concentrations of Ang II in tissue supernatants were determined using a commercially available ELISA kit (Elabscience Biotechnology Co., Ltd., Wuhan, China). All procedures were performed according to the manufacturer’s instructions. Absorbance was measured at 450 nm using a Multiskan GO microplate reader (Thermo Fisher Scientific, software version 1.00.40).

#### Western blot analysis

Protein concentrations in lysates from the Ctrl and DD groups were determined using a BCA Protein Assay Kit (Thermo Scientific, Waltham, MA, USA; Cat. No. 23227) according to the manufacturer’s instructions. Protein samples were mixed with NuPAGE LDS Sample Buffer (Invitrogen, Carlsbad, CA, USA; Cat. No. NP0007) and NuPAGE Sample Reducing Agent (Invitrogen, Carlsbad, CA, USA; Cat. No. NP0009), heated at 97 °C for 5 min, and 30 μg of total protein was loaded onto 4–12% sodium dodecyl sulfate–polyacrylamide gels (SDS–PAGE). Proteins were subsequently transferred onto polyvinylidene fluoride (PVDF) membranes (Merck Millipore, Burlington, MA, USA; Cat. No. IPVH00010). Membranes were blocked using the WesternBreeze Chemiluminescent Kit (Invitrogen, Carlsbad, CA, USA; Cat. No. WB7104) and incubated overnight at 4 °C with the following primary antibodies: mouse monoclonal anti-GAPDH (Invitrogen, Carlsbad, CA, USA; Cat. No. AM4300; 1:3000), rabbit polyclonal anti-AT2R (Thermo Fisher Scientific, Rockford, IL, USA; Cat. No. PA3-210; 1:500), and goat polyclonal anti-AT1R (Novus Biologicals, Centennial, CO, USA; Cat. No. NB100-57073; 1:500). Following washing, membranes were incubated with the appropriate secondary antibodies, and immunoreactive bands were visualized using the WesternBreeze Chemiluminescent Kit. Images were acquired using a Bio-Rad Gel Documentation System (Bio-Rad Laboratories, Hercules, CA, USA).

### *In vitro* contractility assessment

The muscularis propria, separated from the mucosa/submucosa, was cut into approximately 2 × 10 mm strips oriented along the circular muscle axis. Colonic muscular strips were submerged in warm (37 °C) aerated Krebs solution in individual 25 mL wells (Radnoti organ bath, AD instruments Pty, AU) anchored by a metallic hook at the lower end and attached by silk suture to a force transducer for isometric recording of muscular activity (PowerLab^®^, AD Instruments Pty). The muscular strips were initially stretched to 1 g and left to equilibrate for 45 minutes before stimulation, replacing the Krebs solution every 15 min to establish spontaneous contractions at a stable frequency.

Bethanechol (BTCH) (Sigma–Aldrich, USA, CAS 590-63-6), a stable muscarinic agonist that mimics the action of acetylcholine, was used as the cholinergic agonist in this study. Based on our previous findings (Pauza et al., 2019), BTCH 10^4^ M was selected as the reference concentration for maximal contraction, consistent with previous reports showing maximal acetylcholine-induced contraction of human sigmoid colonic muscle at 10^4^ M (Golder et al., 2003). BTCH-induced maximal contraction at 10^-4^ M was compared between the Ctrl and DD groups.

Cumulative concentration–response curves for Ang II 10^-10^–10^-6^ M (Sigma–Aldrich, USA, CAS 4474-91-3) were constructed to determine the concentration eliciting the maximal contractile response and to calculate the half-maximal effective concentration (EC_50_). As 10–^6^ M Ang II produced the maximal contractile response, this concentration was selected for the final comparative experiments. Ang II-induced contractions in the Ctrl and DD groups were expressed as a percentage of the maximal BTCH-evoked contraction.

In comparative experiments, Ang II (10^-6^ M) was added for 5 min to elicit the maximal contractile response and to determine the mean contractile response over the 5-min contraction period. In parallel reservoirs, muscle strips were preincubated with losartan 10–^7^ M (Sigma–Aldrich, USA, CAS 124750-99-8), PD123319 10^-7^ M (Sigma–Aldrich, USA, CAS 136676-91-0), or tetrodotoxin (TTX) (Carl Roth, DE, 4368-28-9) 10^-7^ M for 15 min before the addition of Ang II 10^-6^ M. After the 5-min Ang II response had been recorded, the tissues were washed three times with fresh Krebs solution, and BTCH 10^-4^ M was subsequently added to determine the maximal reference contraction of each muscle strip. Each muscle strip was exposed to only one treatment condition, and all strips within a single experimental run were obtained from the same individual.

### Data analysis statistics

The Shapiro–Wilk test was used to assess data normality. Depending on the distribution of the data, comparisons between experimental groups and treatment conditions were performed using either an unpaired Student’s t-test with Welch’s correction for normally distributed data or the Mann–Whitney U test for non-normally distributed data. These tests were applied, as appropriate, to patient characteristics (**Table 1**), BTCH- and Ang II-induced contractile responses, the effects of losartan, tetrodotoxin (TTX), and PD123319 on Ang II-induced contractions, and Ang II, AT1R, and AT2R expression levels. Statistical analyses were performed using SPSS for Windows (version 23.0; IBM Corp., Armonk, NY, USA) and GraphPad Prism (version 9.0; GraphPad Software, San Diego, CA, USA). A two-sided P value < 0.05 was considered statistically significant.

**Table 1:** Patients’ characteristics.

Patient characteristics	DD (n = 16)	Ctrl (n = 16)	*P*
Age (Mean ± SD)	61.1 ± 14.9	66.4 ± 9.3	0.24
Sex, n (%)			
Male	6 (37.5)	9 (56.3)	0.48
Female	10 (62.5)	7 (43.8)	
BMI, median (IQR)	26.4 (23.7 – 28.9)	25.1 (23.1 – 28.8)	0.85
Diverticulosis grade, n (%)			
None	–	16 (100)	
0-10	4 (24.8)	–	
11-100	11 (68.9)	–	
>100	1 (6.3)	–	
Indications for operation, n (%)			
Diverticulosis	9 (56.3)	–	
Cancer	2 (12.5)	16 (100)	
Stoma occlusion	5 (31.2)	–	
Localization of the tissue, n (%)			
Sigmoid colon	10 (62.5)	8 (50.0)	
Descending colon	5 (31.2)	3 (18.8)	
Transverse colon	1 (6.3)	5 (31.3)	
Preoperative laboratory parameters (Mean ± SD)			
Hemoglobin (g/L)	132.1 ± 18.8	111.6 ± 27.0	0.03
Leucocyte count (×10^9^/L)	6.5 ± 2.1	6.0 ± 1.8	0.56
Neutrophil count (×10^9^/L)	4.0 ± 1.7	4.2 ± 1.5	0.75
Lymphocyte count (×10^9^/L	2.0 ± 1.9	1.3 ± 0.5	0.23
Thrombocytes (×10^9^/L)	262.5 ± 95.1	271.9 ± 94.1	0.80
Urea (mmol/L)	5.3 ± 3.0	4.4 ± 1.5	0.41
Potassium (mmol/L)	4.2 ± 0.4	4.2 ± 0.4	0.71
Sodium (mmol/L)	138.1 ± 3.2	139.6 ± 2.7	0.16
Creatinine (µmol/L)	63.7 ± 19.1	63.4 ± 13.3	0.96
C-Reactive Protein (mg/L), median (IQR)	10.5 (5.0 – 20.0)	5.0 (2.1 – 33.6)	0.28

## Results

**Table 1** summarizes the demographic, clinical and laboratory characteristics of the study population. No significant differences were observed between the DD and Ctrl groups regarding age, sex, BMI, CRP, leukocyte count, neutrophil count, lymphocyte count, thrombocyte count, urea, potassium, sodium or creatinine concentrations. Hemoglobin concentration was significantly higher in the DD group than in the Ctrl group (132.1 ± 18.8 and 111.6 ± 27.0 g/L, respectively; *P* = 0.03). Among patients in the DD group, the majority had 10–100 diverticula (n = 11, 68.9%). Diverticulosis was the most frequent indication for surgery (n = 9, 56.3%), and the sigmoid colon was the most common site of diverticula (n = 10, 62.5%).

### Localization of AT1 and AT2 receptors in colonic muscular tissue

Immunohistochemical analysis showed that AT2R are mostly abundant in the neural tissue (**Figures 1A–C, G–I**). Some neurons of myenteric ganglia (**Figures 1A–F**) are positive to AT2R, also nerve fibers laying between both circular and longitudinal muscle layers are positive to AT2R (**Figures 1G–I**). AT2R immunoreactivity was detected in HuC/HuD-positive myenteric neurons in both the DD and Ctrl groups but appeared more intense in DD specimens.

**Figure 1:**
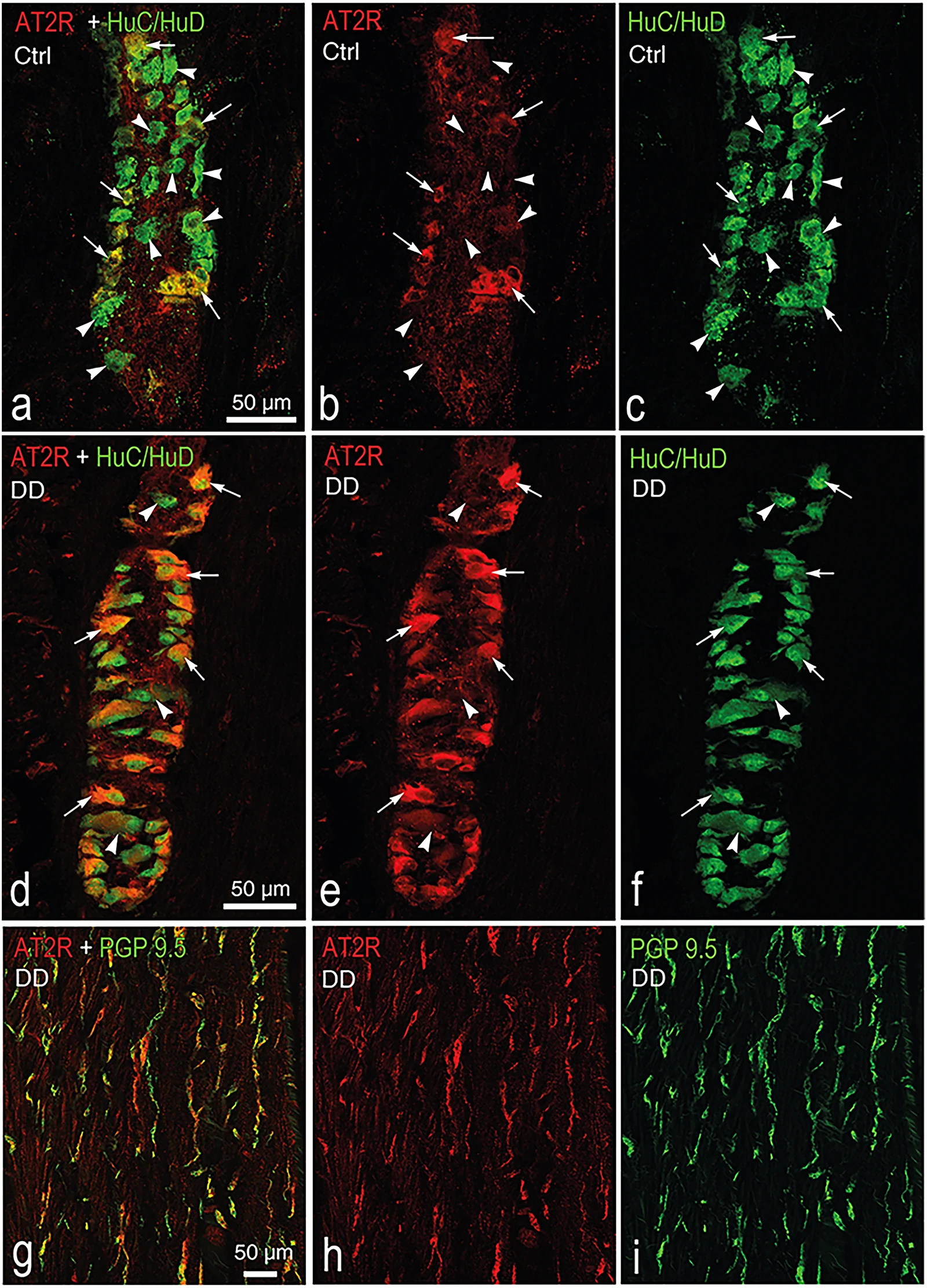
Representative images showing AT2R localization in the human sigmoid colon muscular tissue. Panels **(A–F)** show section of the myenteric ganglia of Ctrl group tissue **(A–C)** and DD **(D–F)**. Some neurons are positive to AT2R (white arrows), but some of them lack positivity, or staining is very weak (white arrowheads). Panels **(A–F)** show the tissue immunolabeled for AT2R (red) and the general neuronal marker HuC/HuD (green). Panels **(G–I)** demonstrate muscular layer, which are highly innervated by nerve fibers positive for PGP 9.5 (green), most of these fibers are positive to AT2R (red).

AT1R was most abundant in the muscular tissue, where it was detected in both circular and longitudinal smooth muscle myocytes, although its distribution was uneven in some areas. AT1R immunoreactivity appeared slightly more intense in the DD group than in controls. (**Figures 2B–E**). In contrast to AT2R, AT1R immunoreactivity was less prominent in PGP9.5-positive myenteric neurons, although it was detected in both DD and control groups. Nevertheless, AT1R immunoreactivity in the myenteric plexus appeared more intense in DD specimens than in controls (**Figures 3A–F**).

**Figure 2:**
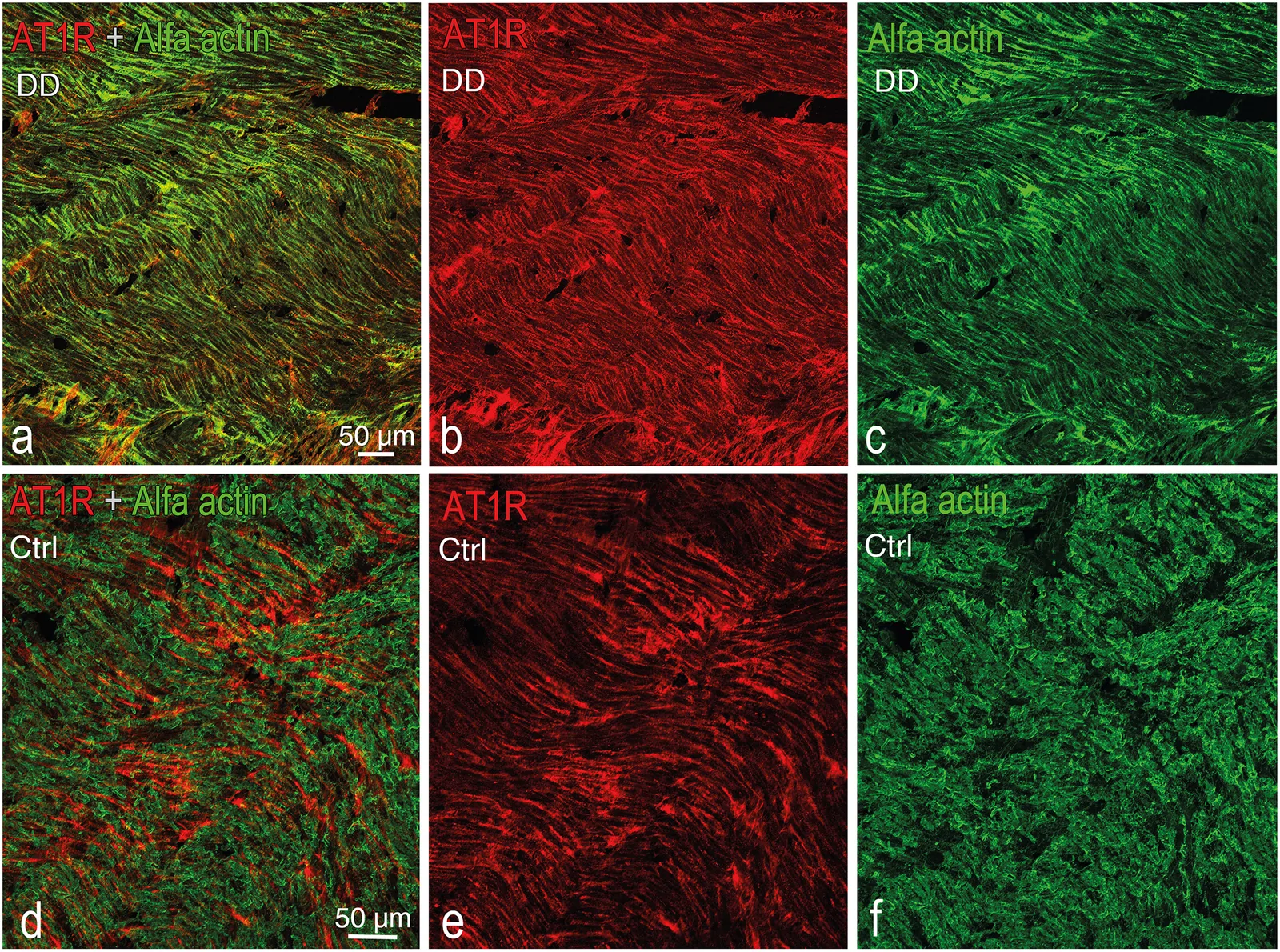
Representative images showing AT1R antibody positivity in the human sigmoid colon muscular tissue. DD patient (panels **(A–C)**) and Ctrl group (panels **(D–F)**) tissue samples were stain to AT1R antibody (red) and anti-alfa actin (green). Note, intensity of the reaction is uneven in some myocytes.

**Figure 3:**
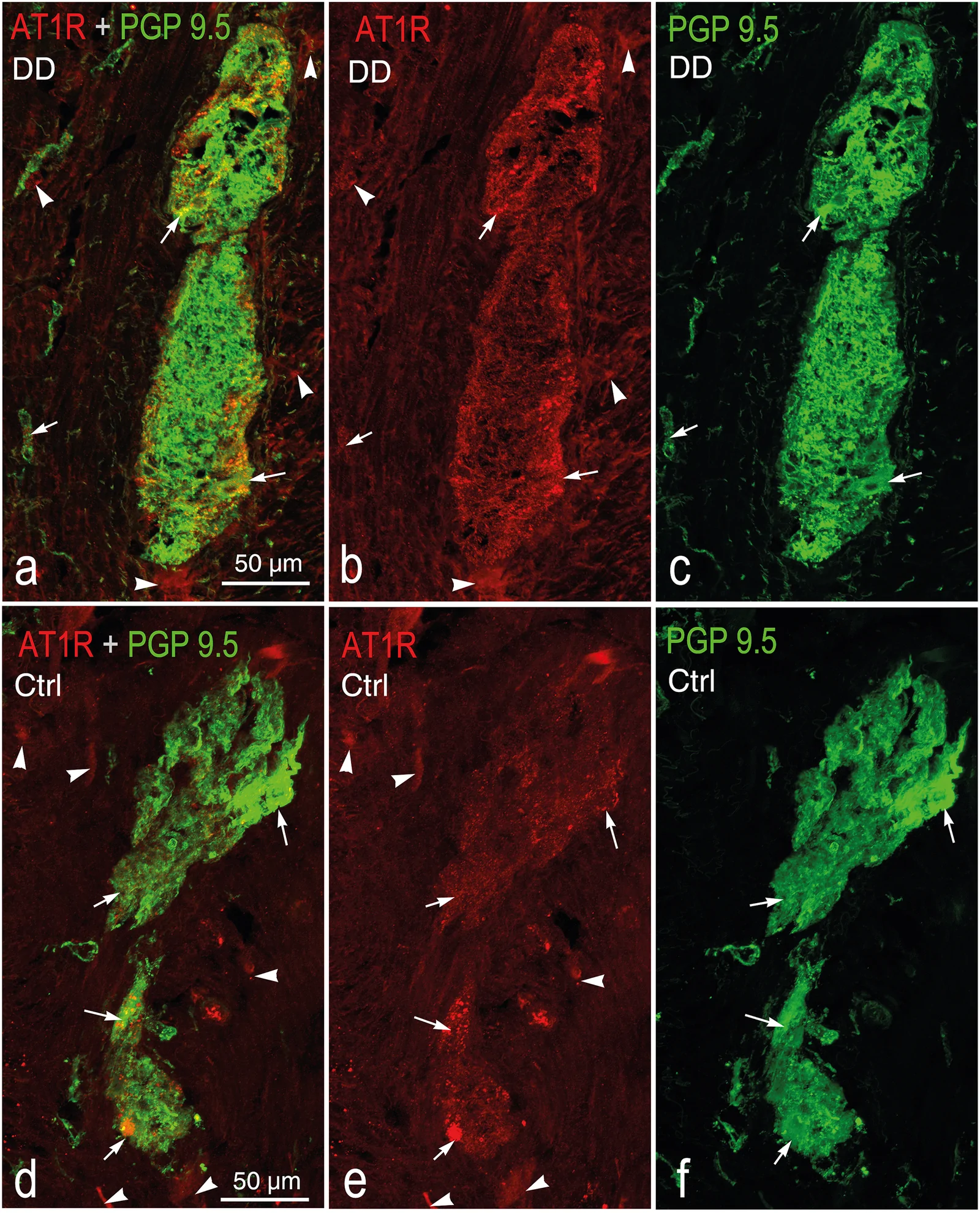
Fluorescence images showing AT1R antibody positivity in the human myenteric plexus. Tissue samples from a DD patient [panels **(A–C)**] and Ctrl group [panels **(D–F)**] were stained with an AT1R antibody (red) and anti-PGP 9.5 antibody (green). Weak AT1R immunoreactivity is visible in the myenteric plexus (white arrows). Muscular layers show AT1R positivity (white arrowheads).

### Ang II levels and mRNA and protein expression of AT1R and AT2R in colonic muscular tissue

Ang II levels in colonic muscular tissue were significantly higher in the DD group than in the Ctrl group (529.7 and 321.2 pg/mL, respectively; **Figure 4A**; P = 0.011). AT1R mRNA expression did not differ significantly between the DD and Ctrl groups (**Figure 4B**). In contrast, AT2R mRNA expression was significantly higher in the DD group than in the Ctrl group (**Figure 4C**; P = 0.007). Similarly to AT1R mRNA expression, AT1R protein expression did not differ significantly between the DD and Ctrl groups (**Figure 4D**). AT2R protein expression was significantly higher in the DD group than in the Ctrl group (4.58 and 2.37, respectively; **Figure 4E**; P = 0.007). The AT1R bands shown in **Figure 4F** appear relatively weak and less intense than the corresponding AT2R bands. However, the quantitative results presented in **Figures 4D, E** reflect background-corrected densitometric values normalized to the corresponding GAPDH signal for each individual sample, rather than visual assessment of band intensity alone.

**Figure 4:**
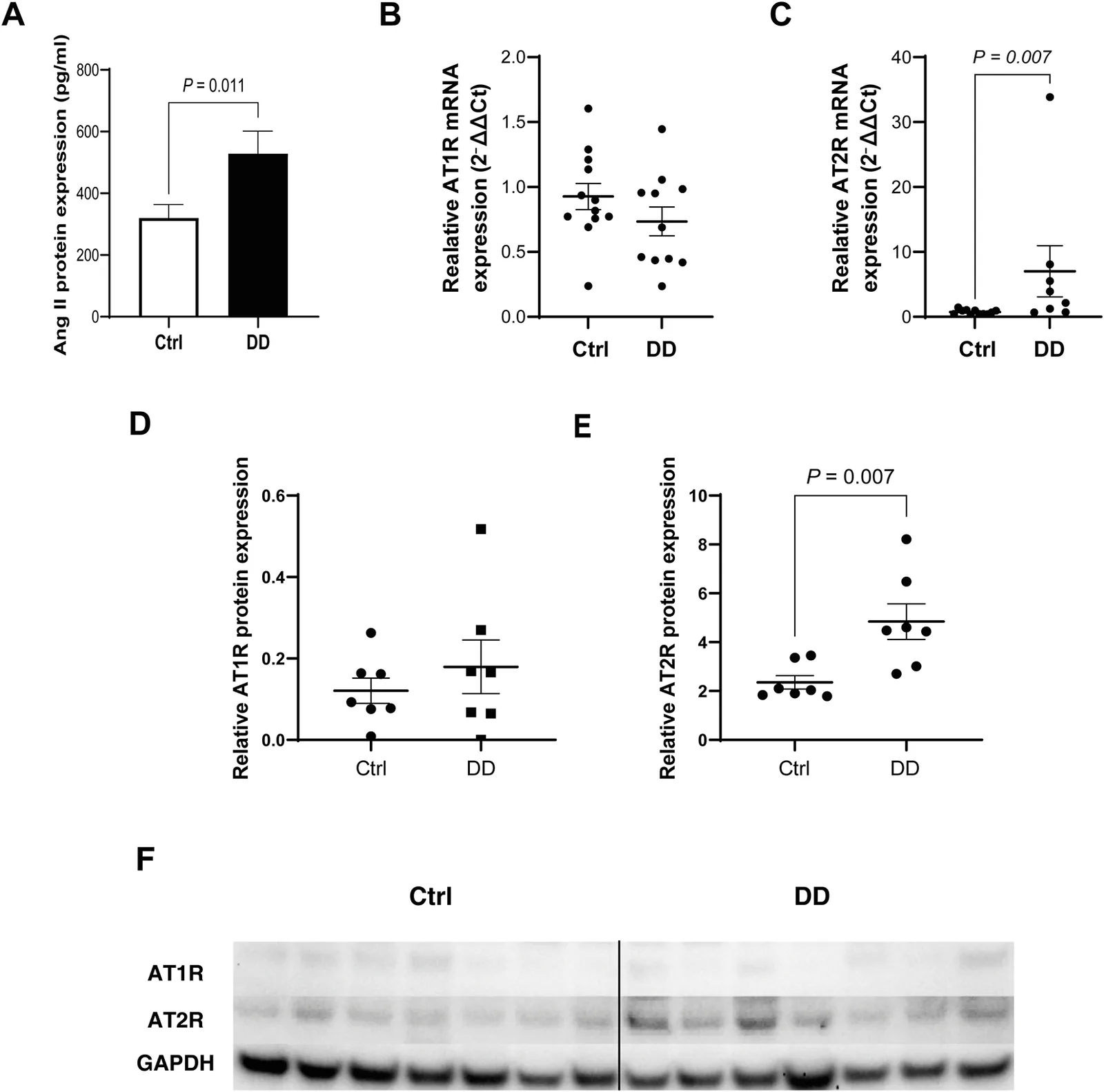
Ang II levels and AT1R and AT2R mRNA and protein expression in colonic muscular tissue. **(A)** Ang II levels determined by ELISA (Ctrl, n = 13; DD, n = 11). **(B)** Relative AT1R mRNA expression determined by RT-qPCR and expressed as fold change (2^-^ΔΔCt) (Ctrl, n = 12; DD, n = 7). **(C)** Relative AT2R mRNA expression determined by RT-qPCR and expressed as fold change (2^-^ΔΔCt) (Ctrl, n = 11; DD, n = 8). **(D)** Densitometric quantification of AT1R protein expression determined by Western blot analysis and normalized to GAPDH (Ctrl, n = 7; DD, n = 7). **(E)** Densitometric quantification of AT2R protein expression determined by Western blot analysis and normalized to GAPDH (Ctrl, n = 7; DD, n = 7). **(F)** Representative Western blot bands for AT1R, AT2R, and GAPDH corresponding to the qualitative analyses shown in panels **(D, E)** Data are presented as mean ± SEM. Student’s t-test or the Mann–Whitney U test was used as appropriate. Statistical significance was considered at P ≤ 0.05.

### Assessment of colonic smooth muscle contractile responses

The cumulative concentration–response curves for Ang II in circular colonic muscle strips are shown in **Figures 5A, B**. Ang II induced concentration-dependent contractions, allowing determination of the EC_50_ (3.25 × 10–^8^ M). The maximal contractile response was achieved at 10–^6^ M, which was subsequently used in all comparative experiments between Ctrl and DD circular muscle strips.

**Figure 5:**
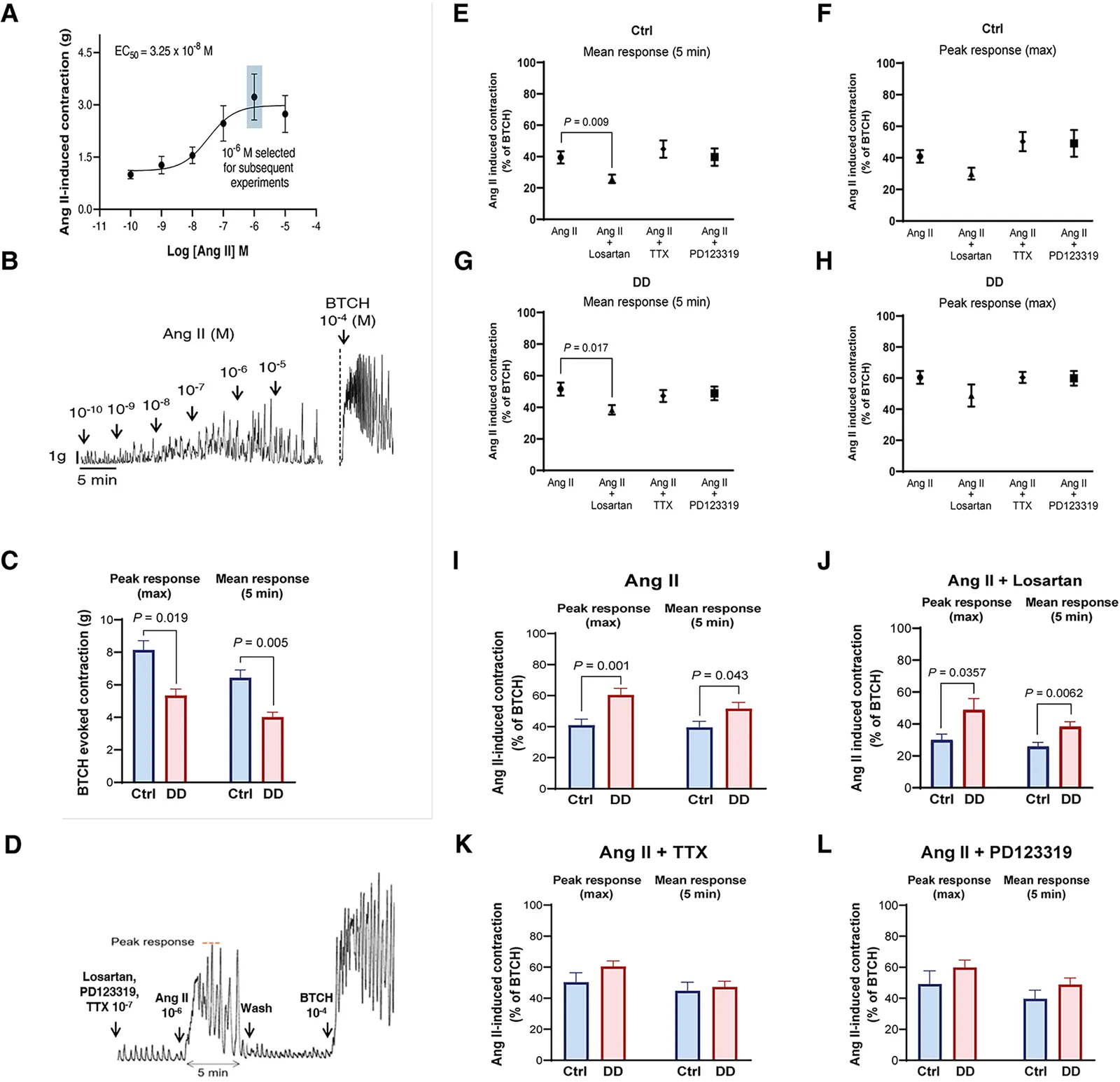
Contractile responses of circular colonic smooth muscle strips from control (Ctrl) and diverticular disease (DD) patients. For the contractility experiments, n denotes the number of individual patients from whom tissue was obtained, whereas N denotes the total number of circular muscle strips analyzed. **(A)** Cumulative concentration–response curve to Ang II demonstrating the EC50 and the maximal response achieved at 10^-6^ M, which was used in subsequent experiments (n = 10, N = 10). **(B)** Representative real-time recording of cumulative Ang II-induced contractions followed by BTCH (10^-4^ M)-induced maximal contraction. **(C)** Peak and mean contractile force during the 5-min period in response to BTCH in Ctrl (n = 10, N = 42) and DD (n = 12, N = 51) circular muscle strips (Mann–Whitney U-test). Peak and mean 5-min contractile responses to bethanechol (BTCH, 10^-4^ M) in circular colonic muscle strips from Ctrl and DD groups, assessed separately to compare cholinergic contractile responsiveness between groups. **(D)** Representative recording illustrating the experimental protocol used for the Ang II experiments shown in panels **(E–L)**. Peak Ang II-induced contractile force was determined as the maximal response (red dashed line), whereas the sustained response was calculated as the mean contractile force over the 5-min recording period. Following washout, BTCH (10^-4^ M) was applied to each individual muscle strip to confirm its contractile responsiveness to cholinergic stimulation and to provide an internal reference for normalization. Ang II-induced responses in panels **(E–L)** were expressed as a percentage of the corresponding BTCH response obtained from the same muscle strip. These BTCH reference responses were independent of the separate BTCH experiments presented in panel **(C, E, F)** Effects of losartan, tetrodotoxin (TTX), and the AT2R antagonist PD123319 on the mean 5-min **(E)** and peak **(F)** Ang II-induced contractile responses in Ctrl circular muscle strips. **(G–H)** Effects of losartan, TTX, and PD123319 on the mean 5-min **(G)** and peak **(H)** Ang II-induced contractile responses in DD circular muscle strips [sample sizes were identical to those shown in panels **(I–L)**]. **(I)** Comparison of peak and mean Ang II-induced contractile responses between Ctrl (n = 10, N = 16) and DD (n = 12, N = 15) circular muscle strips. **(J)** Comparison of Ang II-induced contractile responses following AT1R blockade with losartan between Ctrl (n = 7, N = 7) and DD (n = 9, N = 10) circular muscle strips. **(K)** Comparison of Ang II-induced contractile responses following TTX treatment between Ctrl (n = 9, N = 9) and DD (n = 10, N = 11) circular muscle strips. **(L)** Comparison of Ang II-induced contractile responses following AT2R blockade with PD123319 between Ctrl (n = 8, N = 9) and DD (n = 11, N = 13) circular muscle strips. Ang II-induced responses are expressed as percentages of the corresponding BTCH-induced maximal contraction. Data are presented as mean ± SEM. Statistical analyses were performed using the Mann–Whitney U-test or unpaired t-test with Welch’s correction, as appropriate. Statistical significance was accepted at *P* ≤ 0.05.

Peak BTCH-induced contraction was significantly lower in DD than in Ctrl circular muscle strips (6.44 g and 8.14 g, respectively; *P* = 0.019). Likewise, the mean contractile force during the 5-min recording period was significantly lower in DD than in Ctrl (4.02 g and 5.34 g, respectively; *P* = 0.005) (**Figure 5C**).

In the Ctrl group, losartan significantly reduced the Ang II-induced mean contractile response over the 5-min recording period (39.43% and 25.76% of BTCH, *P* = 0.009; **Figure 5E**. In contrast, neither tetrodotoxin (TTX) nor the AT2R antagonist, PD123319 significantly altered Ang II-induced contractions.

Similarly, in the DD group, losartan significantly reduced the mean 5-min contractile response (51.51% and 38.40% of BTCH, P = 0.017; **Figure 5G**), whereas no significant differences were observed following treatment with TTX or PD123319.

Comparison between groups demonstrated that Ang II alone elicited significantly greater contractions in DD than in Ctrl circular muscle strips (**Figure 5I**). The peak response reached 60.47% of BTCH in the DD group and 40.91% in the Ctrl group (*P* = 0.001), whereas the mean 5-min response was 51.51% and 39.43%, respectively (*P* = 0.043). Following AT1R blockade with losartan, Ang II-induced contractions remained significantly greater in the DD group than in Ctrl tissues (**Figure 5J**). The peak response reached 48.82% of BTCH in DD and 29.98% in Ctrl (*P* = 0.0357), while the mean 5-min response was 38.40% and 25.76%, respectively (*P* = 0.0062).

In contrast, no significant differences between groups were observed following treatment with TTX or PD123319 (**Figures 5K, L**).

## Discussion

Patient characteristics were generally comparable between the DD and control groups, with the only significant difference being hemoglobin concentration. Although previous studies by Tomizawa M. et al. and Lee K. et al. reported lower hemoglobin levels in patients with DD, this has been attributed primarily to diverticular bleeding (Lee et al., 2011; Tomizawa et al., 2015). In contrast, our cohort consisted exclusively of patients with uncomplicated DD, and the mean hemoglobin concentration (132.1 ± 18.8 g/L) was within the normal range, indicating that clinically relevant bleeding was unlikely. The lower hemoglobin concentration observed in the control group (111.6 ± 27.0 g/L) is more likely attributable to anemia associated with colorectal cancer, which commonly results from chronic gastrointestinal blood loss (Chardalias et al., 2023). Therefore, the observed difference in hemoglobin is unlikely to reflect DD itself.

Furthermore, our findings (**Table 1**) showed that the sigmoid colon was the predominant site of diverticula consistent with previous studies reporting the sigmoid colon as the most affected segment in DD (Zollinger, 1968; Hughes, 1969; Peery et al., 2016). Moreover, previous studies suggest that recurrence rates after successful non-operative treatment of diverticulitis are higher in patients with sigmoid diverticulitis than in those with diverticulitis involving other colonic segments (Sung et al., 2022). The susceptibility of the sigmoid colon to diverticular formation may be explained, in part, by its relatively narrow lumen (Sadahiro et al., 1992), which requires stronger circular muscle-mediated segmental contractions and results in higher intraluminal pressures (Painter et al., 1965). In addition, increased colonic wall thickness in DD may not solely represent structural remodeling but may also reflect increased smooth muscle contraction (Wiesner et al., 2002). In this context, our finding that Ang II enhances circular smooth muscle contractility provides further evidence that increased circular muscle activity may contribute to the elevated intraluminal pressure and subsequent mucosal outpouching characteristic of DD.

In the present study, AT1R immunoreactivity was predominantly localized to the smooth muscle layer, although weak expression was also detected in the myenteric plexus, where it appeared more pronounced in the DD group. A similar distribution has been reported previously, with AT1R immunoreactivity detected in both the smooth muscle layers and the myenteric plexus (Mastropaolo et al., 2015). The stronger neuronal immunoreactivity observed in the DD group may be related to the overall upregulation of the RAS, which has been linked to increased inflammation in DD (He et al., 2019; Tursi and Elisei, 2019). Nevertheless, our functional findings demonstrated that tetrodotoxin did not attenuate Ang II-induced contractions, indicating that the contractile response is mediated primarily through direct activation of smooth muscle rather than enteric neural pathways. These findings are consistent with previous studies demonstrating that, despite the presence of AT1R in enteric neurons, Ang II-induced intestinal contraction results predominantly from direct action on the musculature rather than stimulation of cholinergic neurotransmission (Wang et al., 2005). Although neither TTX nor AT2R blockade significantly affected Ang II-induced contractions in the present study, previous studies in rat anococcygeus smooth muscle demonstrated an AT2R-mediated, tetrodotoxin-sensitive neurogenic relaxant response involving the nNOS/sGC pathway (De Godoy et al., 2004). In addition, another study reported that TTX reduced Ang II-induced contractions in the mouse proximal colon and completely abolished them in the distal colon (Mastropaolo et al., 2013). These discrepancies may reflect tissue- and species-specific differences in Ang II signaling, together with regional heterogeneity in the functional role of AT2R among smooth muscle from different intestinal segments.

In the present study, both Ang II levels and AT2R protein expression were significantly increased in the colonic muscular tissue of patients with DD compared with controls. At the transcriptional level, AT2R mRNA expression was also significantly increased in DD, consistent with the observed increase in AT2R protein expression. In contrast, AT1R mRNA and protein expression did not differ significantly between the DD and Ctrl groups. Therefore, the present findings do not provide sufficient evidence for altered AT1R protein and mRNA expression in DD, and further studies are needed to clarify this issue.

Previous studies using isolated smooth muscle cells have demonstrated that increasing Ang II concentrations induce AT1R internalization from the plasma membrane to the cytosol, representing a physiological receptor trafficking mechanism that may limit excessive receptor activation rather than causing receptor inactivation (De Godoy and Rattan, 2006). In the present study, AT1R protein expression did not differ significantly between the DD and control groups, and AT1R expression was relatively weak in both groups. These findings are compatible with, but do not demonstrate, altered AT1R trafficking. Because our analyses were performed on full-thickness muscular tissue and did not distinguish between membrane-associated and intracellular receptor pools, no direct conclusions regarding AT1R internalization can be drawn. Nevertheless, the increased Ang II levels and enhanced contractile responses observed in DD suggest that altered Ang II signaling may occur without an increase in AT1R expression. Although AT1R protein expression was not significantly increased in DD, the contractility experiments demonstrated that blockade of AT1R reduced the contractile response to Ang II, supporting a functional contribution of AT1R to Ang II-mediated contraction. However, it remains unclear whether AT1R trafficking or recycling is altered in DD.

In contrast to AT1R, AT2R immunoreactivity was predominantly localized to the myenteric plexus and nerve fibers between the circular and longitudinal muscle layers, with no detectable expression in smooth muscle cells. AT2R immunoreactivity appeared more intense in the DD group than in controls, corresponding to the increased AT2R protein expression detected in the muscular tissue. Since protein expression was quantified in the entire muscular layer, which also includes the myenteric plexus and associated nerve fibers, the increased AT2R expression likely reflects its enhanced neuronal expression. A similar neuronal localization of AT2R has previously been reported in human jejunal muscular tissue, where AT2R was predominantly detected in myenteric nerves but not in smooth muscular layer (Spak et al., 2008).

One of the principal findings of the present study was that losartan significantly attenuated the mean 5-min Ang II-induced contractile response in both Ctrl and DD circular muscle strips. In addition, comparison between groups demonstrated that, following AT1R blockade with the same concentration of losartan (10^-7^ M), both peak and mean Ang II-induced contractile responses remained significantly greater in DD than in Ctrl tissues, suggesting reduced responsiveness to AT1R blockade in DD. A limitation of the present study is that Ang II- and losartan-treated contractile responses were obtained from independent tissue samples derived from different patients rather than paired preparations. Therefore, the magnitude of losartan-induced inhibition could not be quantified directly within the same tissue, and comparisons were limited to between-group differences in the residual contractile responses following AT1R blockade. However, this finding is consistent with the increased local Ang II levels and the predominant localization of AT1R within the smooth muscle layer, both of which may contribute to the enhanced contractile response. Nevertheless, the role of AT1R expression remains unclear, as the present study did not demonstrate a significant increase in AT1R protein levels in the DD group. Similar inhibitory effects of losartan on Ang II-induced contractions have been reported previously. However, in contrast to our ex vivo experiments, losartan was administered orally *in vivo* to spontaneously hypertensive rats, resulting in adaptive alterations in intestinal smooth muscle contractility following chronic AT1R blockade (Patten and Abeywardena, 2017). Unlike the chronic oral losartan treatment used in the spontaneously hypertensive rat model, losartan was applied acutely *in vitro* to colonic tissue obtained from patients who were not receiving angiotensin receptor blockers, ACE inhibitors, or other medications affecting the RAS. Therefore, the enhanced contractile responses observed in DD most likely reflect disease-related activation of the local RAS rather than adaptations induced by chronic pharmacological blockade.

Another important finding of the present study was that BTCH-induced circular smooth muscle contractions were significantly reduced in the DD group compared with, indicating impaired cholinergic contractile function of the circular muscle. BTCH-induced contractions were assessed separately to directly compare cholinergic contractile responsiveness between the Ctrl and DD groups and to determine whether previously reported alterations in cholinergic function in DD were also present in our study population. These BTCH measurements were not used for normalization of the Ang II-induced contractile responses. The reduced responsiveness to cholinergic stimulation is consistent with previous studies demonstrating that DD is associated with local alterations in electrical activity, leading to changes in segmental contractile responses (Huizinga et al., 1999). Furthermore, reduced choline acetyltransferase activity together with upregulation of M3 muscarinic receptors has been reported in the circular muscle of patients with DD, resulting in increased sensitivity to exogenous acetylcholine despite impaired cholinergic innervation (Golder et al., 2003).

In the Ang II contractile response experiments, each individual muscle strip was exposed to BTCH at the end of the experiment, and its Ang II-induced response was normalized to the BTCH-induced contraction obtained from the same strip. This normalization approach is commonly used *in vitro* contractility studies of gastrointestinal smooth muscle (Ewert et al., 2006; Mastropaolo et al., 2013; Malinauskas et al., 2014). BTCH was used as an internal contractile reference because it elicited a robust muscarinic receptor-mediated contraction exceeding the maximal response induced by Ang II. The Ang II concentration response curve reached its maximal contractile response at 10^-6^ M, and increasing the Ang II concentration further did not produce a greater contraction. This maximal Ang II response remained below the contraction elicited by BTCH at 10^-4^ M. Thus, BTCH provided a suitable reference against which Ang II-induced contractile responses could be expressed.

The opposing changes observed in DD reduced cholinergic contractile responsiveness together with enhanced relative responsiveness to Ang II may therefore indicate a shift in the regulation of circular smooth muscle contractility, whereby Ang II becomes a more prominent regulator as cholinergic control is impaired, potentially promoting abnormal circular muscle activity in DD.

## Conclusions

The present study demonstrates altered regulation of colonic circular smooth muscle contractility in DD, characterized by enhanced responses to Ang II and reduced responses to cholinergic stimulation. The distinct localization of AT1R predominantly in smooth muscle and AT2R in the myenteric plexus and nerve fibers suggests differential roles of Ang II signaling at muscular and neural levels. These alterations may promote abnormal circular smooth muscle activity and increased intraluminal pressure, thereby being involved in the pathogenesis of DD. Although the present functional findings support a predominant role of AT1R in Ang II-induced contraction, the potential neural role of AT2R requires further investigation.

## Data Availability

The original contributions presented in the study are included in the article/supplementary material; further inquiries can be directed to the corresponding author.
